# Fatigue and neuromuscular function in long COVID: A one-year follow-up study

**DOI:** 10.1371/journal.pone.0332242

**Published:** 2025-09-24

**Authors:** Isabella da Silva Almeida, Leandro Gomes de Jesus Ferreira, Marco Aurélio Vaz, Gerson Cipriano Junior, Monique Alves de Resende, Denis Cesar Leite Vieira, Rochelle Rocha Costa, Nicolas Babault, Rita de Cássia Marqueti, João Luiz Quagliotti Durigan

**Affiliations:** 1 Laboratory of Muscle and Tendon Plasticity, Graduate Program in Rehabilitation Science, Faculdade de Ceilândia, Universidade de Brasília, Brasília, Distrito Federal, Brazil; 2 Graduate Program in Physical Education, Physical Education Department, Universidade de Brasília, Brasília, Distrito Federal, Brazil; 3 Laboratory of Molecular Analisys, Graduate Program in Rehabilitation Science, Faculdade de Ceilândia, Universidade de Brasília, Brasília, Distrito Federal, Brazil; 4 Exercise Research Laboratory, Federal University of Rio Grande do Sul, Porto Alegre, Brazil; 5 Graduate Program in Rehabilitation Science, Rehabilitation Science Department, Faculdade de Ceilândia, Universidade de Brasília, Brasília, Distrito Federal, Brazil; 6 Strength and Conditioning Research Laboratory, College of Physical Education, University of Brasília, Brasília, Brazil; 7 Centre d’Expertise de la Performance, INSERM U1093 CAPS, Sports Science Faculty, University of Burgundy, Dijon, France; University of Utah, UNITED STATES OF AMERICA

## Abstract

**Background:**

Long COVID has emerged as a significant complication of SARS-CoV-2 infection. However, the long-term neuromuscular consequences of this condition, particularly one-year post-infection, remain poorly understood. This study aimed to determine the mechanisms of fatigue by comparing perceived fatigue, objective fatigability, functionality, muscle architecture, and electrical neuromuscular function in participants who had suffered severe or moderate COVID‑19 one-year post-infection with a healthy control group.

**Methods and Findings:**

This longitudinal observational study followed participants for one-year. The assessments were conducted at the Laboratory of Muscle and Tendon Plasticity at the University of Brasília, Brazil. Participants who had suffered moderate or severe SARS-CoV-2 infection were compared to a control group. A baseline assessment was initially conducted (21–30 days post-symptoms onset or post-hospital discharge), followed by second (31–90), third (91–180), and fourth (181–360) assessments. Perceived fatigue, objective fatigability, functionality, muscle architecture, and electrical neuromuscular function were analyzed. The study included 30 controls (46.53 [42.10–51.43] years; 13 men [43.33%]), 22 moderate cases (38.27 [33.96–43.13] years; 10 men [45.45%]), and 18 severe cases (50.83 [45.19–57.18] years; eight men [44.44%]). Severe participants exhibited higher perceived fatigue in all assessments than the control group and at baseline and in assessment 4 compared to moderate cases, in addition to a lower torque and torque-time integral in all assessments of objective fatigability analysis compared to the other groups. The severe group also demonstrated reduced functionality, impaired muscle architecture (characterized by increased echogenicity), and higher chronaxie values in the electrical neuromuscular function assessment. Participants with moderate COVID‑19 exhibited alterations in perceived fatigue, reduced torque, and lower TTI, electrical neuromuscular function, and muscle architecture, particularly at baseline.

**Conclusions:**

Severe participants continued to experience significant perceived fatigue even one-year post-infection, suggesting a slower recovery trajectory, that contributed to increased fatigability throughout the follow-up period. These results emphasize the role of musculoskeletal and neural mechanisms in post-COVID‑19 fatigue, highlighting the need for targeted, mechanism-based rehabilitation strategies.

**Trial registration:**

NCT04961255

## Introduction

Long COVID has emerged as a severe complication following SARS-CoV-2 infection. This condition can develop after both severe and mild cases of the initial COVID‑19 illness and is characterized by persistent symptoms that last for more than 12 weeks beyond the acute infection phase [1]. Long COVID prevalence rates range from 10% to 50%, with long-term sequelae reported in up to 50% of cases [1]. Symptoms, including fatigue, can persist for weeks or months, affecting up to 63% of patients, even those with moderate symptoms, impacting daily activities and a return to work [2–7]. Four years into the COVID‑19 pandemic, long-term complications remain a major public health concern [3,8]. Long COVID results in prolonged illness for patients and creates significant health and socio-economic challenges, including strained rehabilitation facilities and long-term workforce losses [9]. Recent research on long COVID highlights complications such as fatigue, muscle weakness, and functional deficits, as well as changes in muscle function and composition, that can persist for up to two years after infection [3–5,8,10–13], with those hospitalized during the acute phase facing a higher risk of death [4].

Previous studies demonstrated that post-COVID fatigue has been linked to skeletal muscle abnormalities, including amyloid deposits, mitochondrial dysfunction, and exercise-induced myopathy that worsens after exertion [11]. Muscle biopsies in post-COVID patients reveal chronic inflammation, capillary damage, and fiber-type shifts [14], suggesting distinct pathophysiological processes compared to other fatigue syndromes [15]. One interesting follow-up study suggests that within a year, measurable improvements in neurophysiological function, as well as in mitochondrial and autonomic parameters, may occur, coinciding with a decline in reported fatigue symptoms [16]. While many studies offer a broad overview of fatigue, the specific factors underlying its persistence, such as muscle strength, neuromuscular activation, muscle architecture, and peripheral nervous system function, have not yet been fully elucidated. Thus, the current study aimed to determine the mechanisms related to fatigue by evaluating and comparing perceived fatigue, objective fatigability, functionality, muscle architecture, and electrical neuromuscular function in a healthy control group and participants who had suffered moderate or severe COVID‑19 one-year post-infection. We hypothesized that disease severity affects the musculoskeletal and neural systems, negatively impacting fatigability and functionality. To our knowledge, this is the longest follow-up cohort study investigating neuromusculoskeletal alterations and muscle fatigability in COVID‑19 survivors of varying disease severities, using objective measures and comparisons with a healthy control group.

## Materials and methods

### Study population

**Fig 1:**
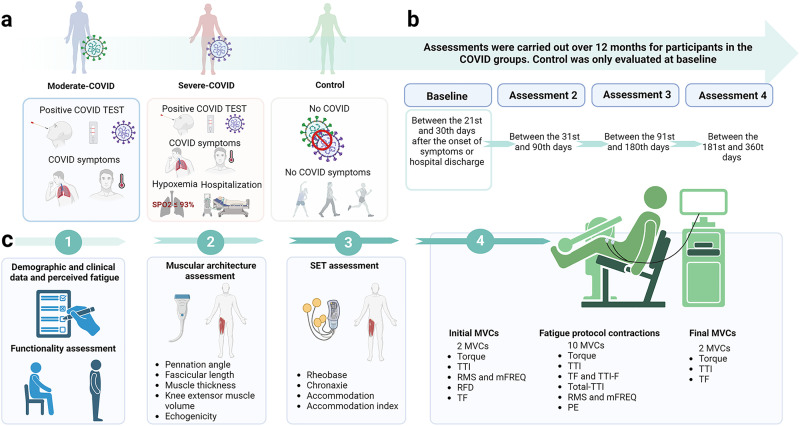
Participants and study flow. a. Participants classification. Moderate-COVID participants were required to present a positive COVID‑19 test. They also needed to exhibit some symptoms, such as a dry cough, runny nose, sore throat, diffuse body pain, and persistent hyperthermia, without hypoxemia. Severe-COVID participants were required to present a positive test for COVID‑19, at least one of the symptoms mentioned above, and hypoxemia requiring hospitalization. Participants without a prior COVID‑19 infection or symptoms were invited to participate in the control group. Exclusions included a history of neuromuscular or orthopedic lower limb issues, pregnancy, active infection, or neurological problems. b. Assessments. c. Procedure and outcomes. Participants received all necessary information and were familiarized with the procedures. Demographic and clinical information, as well as the fatigue severity scale (FSS), were obtained. The participant performed the sit-to-stand test (30s-STS) and was positioned on the stretcher to perform the muscle ultrasound and stimulus electrodiagnostic test (SET) assessments at rest. The participant was then positioned on the isometric dynamometer where a warm-up was performed, and the fatigue assessment was carried out with torque and electromyography analysis followed by the fatigue protocol analysis. For this, the participants performed 2 initial maximal voluntary contractions (MVC), followed by 10 MVCs (fatigue protocol), and concluded with 2 final MVCs. In the initial 2 MVCs, the torque, torque-time integral (TTI), rate of force development (RFD), and electromyography including root mean square (RMS) and median frequency (mFREQ) were analyzed. In addition, the torque fatigability (TF) was calculated using the torque of the initial 2 MVCs and 2 final MVCs. During the fatigue protocol, the torque, TTI, total-TTI, torque fatigability (TF), TTI fatigability (TTI-F), RMS, mFREQ, and perceived exertion (PE) were analyzed.

This one-year longitudinal study was approved by the University of Brasília ethics committee (CAAE: 45043821.0.0000.8093) and registered at clinicaltrials.gov (NCT04961255). Written informed consent was obtained from all participants. The participants were included in the study from September 27, 2021, to May 22, 2023. Participants aged 18–80 with a history of moderate (moderate-COVID – n = 22) or severe (severe-COVID – n = 22) SARS-CoV-2 infection were recruited from local clinics and hospitals [17,18]. Participants without prior COVID‑19 infection or symptoms were invited to participate in the control group (n = 30). Moderate-COVID participants were required to present a positive COVID‑19 test confirmed by a molecular test, serological test reactivity to antibodies, or remote laboratory test. Additionally, they needed to exhibit some symptoms, such as a dry cough, runny nose, sore throat, diffuse body pain, and persistent hyperthermia, without hypoxemia. Severe-COVID participants were required to present a positive test for COVID‑19, at least one of the symptoms mentioned above, and hypoxemia (oxygen saturation – SPO2 ≤ 94%, and/or respiratory rate ≥ 30 breaths per minute, and/or lung infiltrates affecting more than 50% of the lung fields) requiring hospitalization [17,18]. Participants without a prior COVID‑19 infection or symptoms were invited to participate in the control group. Exclusions included a history of neuromuscular or orthopedic lower limb issues, pregnancy, active infection, and neurological problems. In addition, before the inclusion of each participant in the control group, they were asked about their history of infections during the pandemic period. Participants were not included in the control group if they reported experiencing symptoms characteristic of COVID‑19 infection or if they had previously tested positive for the disease (Fig 1A).

### Study design

All participants followed the same protocol, with four laboratory visits over one year to perform the assessments, except the control group, which was assessed only at baseline to prevent any influence of a SARS-CoV-2 infection during follow-up. The first assessment was considered as baseline and was performed 21–30 days after the occurrence of symptoms for moderate-COVID and after hospital discharge for severe-COVID, followed by the second (31st to 90th days), third (91st to 180th days), and fourth assessments (181st to 360th days – Fig 1B).

### Demographic and clinical data and perceived fatigue

Demographic and clinical data were obtained, and perceived fatigue was assessed using the Fatigue Severity Scale questionnaire in each assessment [19].

### Assessment of functionality, muscle architecture and electrical neuromuscular abnormalities

The 30-second sit-to-stand test (30s-STS) was used to assess functionality, starting with two to three slow repetitions for familiarization. A score of 0 was given if the participant did not perform any repetitions [20]**.** The muscle architecture assessment involved capturing muscle images using B-mode ultrasound (M-Turbo®, Sonosite, Bothwell, WA, USA; Frequency: 10MHz, Depth: 6 cm) [21,22]. The rectus femoris (RF), vastus medialis (VL), and vastus lateralis (VM) were evaluated as previously described [22]. The pennation angle (PA), fascicular length (FL), muscle thickness (MT), and echogenicity were evaluated with excellent intra- and inter-rater reliability for most quadriceps femoris measurements [21]. The muscle volume (MV) of the knee extensor muscles was calculated as proposed by Miyatani et al [23].

The stimulus electrodiagnostic test (SET) was used to identify electrical neuromuscular abnormalities. Rheobase, chronaxie, accommodation, and the accommodation index were obtained for RF, VL, and VM muscles [24] using a pulse generator (Dualpex 071, Quark Medical LTDA, Piracicaba, Brazil) with high to very high intra- and inter-rater reliability [24,25] (Fig 1C).

### Fatigability assessment

Muscle force was assessed objectively through torque production and electromyography activity, measured using surface electromyography. Participants sat on an isometric dynamometer (IsoSystem 2.0, Cefise, SP, Brazil) with their limb secured at 60 degrees of knee flexion [26,27]. Two self-adhesive electrodes (2.5 cm^2^; NeuroPlus, Nissha Medical Technologies, NY, USA) were placed ~1 cm apart over the middle portion of the belly of the RF, VL, and VM muscles [28].

The testing began with a warm-up of three submaximal voluntary isometric contractions (10 seconds each) and a 1-minute rest between contractions. For the fatigue assessment, participants performed two initial maximal voluntary contractions (MVCs) of 10 seconds each, followed by a 1-minute rest. They then completed 10 successive MVCs, each lasting 10 seconds, with approximately 6 seconds of rest between each contraction. Finally, participants performed two further MVCs, each lasting 10 seconds [29,30]. The torque-time integral (TTI) was defined as the area under the torque-time curve [31]. The total-TTI was calculated by summing the TTI of the 10 MVCs to quantify the total work during the fatigue protocol [31]. The rate of force development (RFD) was calculated from the two initial MVCs, as the maximum slope of the force-time curve. The onset for force was defined as the time instant when the force exceeded 7.5 N.m [32]. In these MVCs, the participants were instructed to perform the test with as much force and as quickly as possible, in an explosive way. The root mean squared (RMS), and median frequency (mFREQ) values were calculated over 500 ms around the maximum torque, including 250 ms before and after the peak [33]. Relative RMS values were normalized to the amplitude of the RMS obtained in the 2 initial MVCs. Perceived exertion (PE), using the Borg scale (6–20) [34], was recorded before starting the fatigue protocol and after each fatigue protocol MVC. Torque fatigability (TF) was quantified as the percentage difference between the initial and final MVCs [35]. The TF and TTI fatigability (TTI-F) were quantified by defining the average value of the initial two MVCs as 100% and expressing the fatigue protocol of MVCs 1, 5, and 10 as a percentage of this average [36] (Fig 1C).

### Statistical analysis

No calculations were made to determine the ideal sample size, considering the lack of previous data. The Generalized Estimating Equations (GEE) method was adopted, using “group” and “assessments” as factors, and “group”, “assessment”, and “contractions” as factors for the fatigue protocol outcomes. The independence model criterion [37] was used to select the best functional correlation structure in the GEE analyses. Model fit was tested for all outcomes considering the moderators “sex”, “age”, and the combination of “sex and age”. Models were adjusted based on the lowest QIC value and **P* *< 0.05 for the tested moderator, with LSD used as a post hoc test. For variables in which the moderator did not yield *P* < 0.05, the model was run without any moderator. To compare categorical variables, the Chi-Square Test was adopted. The level of statistical significance adopted was α < 0.05 and statistical analyses were performed with SPSS 22.0 (IBM Corporation, Armonk, NY, USA).

## Results

In total, 190 assessments were conducted from September 2021 to October 2023. Assessments included 22 moderate-COVID, 18 severe-COVID, and 30 control participants. For torque, electromyography, and fatigue assessments, 15 severe-COVID participants completed the baseline test, as 3 were unable to perform the fatiguing task.

### Demographic and clinical data

Participants in the moderate-COVID group were younger than those in both the severe-COVID (p = 0.001) and control groups (p = 0.018). The severe-COVID had higher body weight (severe-COVID vs moderate-COVID [p = 0.029]; severe-COVID vs control [p = 0.003]), and body mass index (severe-COVID vs moderate-COVID [p < 0.001]; severe-COVID vs control [p < 0.001]), and more comorbidities (p < 0.05). They were also less physically active before and during assessments (p < 0.05) and more individuals in this group had undergone post-COVID physiotherapy (p < 0.001). The control had a longer smoking cessation period among ex-smokers (control vs severe-COVID [p = 0.012]; control vs moderate-COVID [p = 0.002]) and received more COVID‑19 vaccine doses (control vs severe-COVID [p < 0.001]; control vs moderate-COVID [p < 0.001], Table 1).

**Table 1:** Characteristics of the participants.

	Groups			
	Control group	Moderate-COVID	Severe-COVID	
	(n = 30)	(n = 22)	(n = 18)	
Characteristics	Mean (CI 95%)	Mean (CI 95%)	Mean (CI 95%)	*P* value
**Sex (number of participants/% of participants)**				
Male	13 (43.33%)	10 (45.45%)	8 (44.44%)	0.988
Female	17 (56.66%)	12 (54.54%)	10 (55.55%)	
**Age (years)**				
Baseline assessment	46.53 (42.10–51.43)	38.27 (33.96–43.13)*	50.83 (45.19–57.18)^#^	0.001
Assessment 2		38.36 (34.04–43.23)*	51.11 (45.52–57.39)^#^	
Assessment 3		38.68 (34.33–43.59)*	51.28 (45.60–57.66)^#^	
Assessment 4		39.18 (34.90–43.99)*	51.89 (46.22–58.25)^#^	
**Weight (kg)**				
Baseline assessment	69.54 (65.07–74.31)	71.28 (64.14–79.21)	83.08 (75.91–90.92)^#^*	0.001
Assessment 2		71.57 (64.50–79.42)	84.89 (77.27–93.25)^a #^*	
Assessment 3		72.32 (65.14–80.28)	85.43 (77.51–94.15)^a #^*	
Assessment 4		73.12 (65.96–81.08)^a, c^	87.49 (79.89–95.81)^a, b, c #^*	
**Height (m)**	1.66 (1.63–1.69)	1.66 (1.62–1.70)	1.61 (1.55–1.66)	0.206
**BMI (kg/m**^**2**^)				
Baseline assessment	24.91 (23.85–26.02)	25.55 (23.51–27.77)	31.94 (29.70–34.35)^#^*	0.001
Assessment 2		25.53 (23.46–27.77)	32.55 (30.34–34.91)^#^*	
Assessment 3		25.89 (23.76–28.20)	32.72 (30.45–35.16)^#^*	
Assessment 4		26.13 (24.10–28.33)^a, b^	33.62 (31.55–35.83)^a, b, c #^*	
**Comorbidities (% of participants)**				
Hypertension	5 (16.66%)	1 (4.54%)	18 (66.66%)^#^	0.001
Diabetes	0 (0%)	1 (4.54%)	7 (38.88%)*	0.001
Dyslipidemia	6 (20.00%)	3 (13.63%)	9 (50.00%)^#^	0.021
Depression	0 (0%)	1 (4.54%)	5 (27.77%)*	0.003
Anxiety	1 (3.33%)	5 (22.72%)	3 (16.66%)	0.102
Panic Syndrome	0 (0%)	1 (4.54%)	2 (11.11%)	0.183
Asthma	0 (0%)	4 (18.18%)	2 (11.11%)	0.062
Hospitalization period (days)	NA	NA	38.38 (27.15–49.62)	NA
Period in ICU (days)	NA	NA	21.44 (13.72–29.16)	NA
**COVID vaccine before infection (n) for moderate and severe-COVID groups and COVID vaccine at the time of evaluation for the Control (% of participants)**				
Yes	28 (93.33%)	22 (100%)	16 (88.88%)	0.298
No	2 (6.66%)	0 (0%)	2 (11.11%)	
Number of doses	3.50 (3.25–3.77)	2.14 (1.89–2.41)*	2.00 (1.72–2.32)*	0.001
**Current smoker (% of participants)**				
Yes	1(3.33%)	1 (4.54%)	0 (0%)	0.677
No	29 (96.66%)	21 (95.45%)	(100%)	
Time (Years)	0.03 (0.00–0.06)	0.02 (0.00 - 0.05)	0.00 (0.00–0.00)	
**Previous smoker (% of participants)**				
Yes	5 (16.66%)	7 (31.81%)	6 (33.33%)	0.323
No	25 (83.33%)	15 (68.18%)	12 (66.66%)	
Time (years)	16.60 (8.40–32.79)	15.85 (11.00–22.85)	27.16 (19.67–37.51)	0.076
Interruption time (years)	24.40 (19.34–30.78)	10.37 (5.45–19.72)*	11.63 (5.77–23.42)*	0.011
**Practice of physical activity before infection and at the time of assessment for the Control group (% of participants)**				
Yes	19 (63.33%)	9 (40.90%)	5 (27.77%)*	0.045
No	11 (36.66%)	13 (59.09%)	13 (72.22%)	
**Practice of physical activity at the time of assessment (% of participants)** ^**$**^				
Baseline assessment				0.001
Yes	19 (63.33%)	8 (36.36%)	3 (16.66%)^#^*	
No	11 (36.66%)	14 (63.63%)	15 (83.33%)	
Assessment 2				
Yes		14 (63.63%)	5 (27.77%)^#^*	
No		8 (36.36%)	13 (72.22%)	
Assessment 3				
Yes		17 (77.27%)	5 (27.77%)^#^*	
No		5 (22.72%)	13 (72.22%)	
Assessment 4				
Yes		14 (63.63%)	4 (22.22%)^#^*	
No		8 (36.36%)	14 (77.77%)	
**Physiotherapy after infection (% of participants)**				
Yes	NA	0 (0%)	13 (72.22%)^#^	0.001
No	NA	22 (100%)	5 (27.77)	

Footnote: NA, not applicable; * = Different from the Control in the respective assessment; # = Different from moderate-COVID in the respective assessment; a = Different from baseline assessment; b = Different from assessment 2; c = Different from assessment 3. (p < 0.05).

^$^For this study, participants who reported being involved in any sporting or conditioning activity, and regularly practicing the modality at least twice a week, were considered to be practicing physical activity [38].

### Perceived fatigue

Perceived fatigue (Table 2, and Data S1 in S2 File) showed a significant interaction between group and assessment (p < 0.001), with higher perceived fatigue in COVID‑19 groups compared to the control (p < 0.05) in all assessments. The severe-COVID displayed greater fatigue than the moderate-COVID group at baseline (p = 0.027) and assessment 4 (p = 0.024). Intragroup comparisons showed a reduction in perceived fatigue at assessments 2, 3, and 4 compared to baseline for moderate-COVID and in assessments 2 and 3 compared to baseline for severe-COVID.

**Table 2:** Perceived fatigue analysis and objective fatigability analysis during knee-extension maximal torque production.

		Baseline assessment	Assessment 2	Assessment 3	Assessment 4	GEE (*p* values)		
	Groups	Mean (95% CI)	Mean (95% CI)	Mean (95% CI)	Mean (95% CI)	Group	Assessment	Group* Assessment
**Perceived fatigue analysis**								
FSS	Control (n = 30)	2.75 (2.33–3.24)				<0.001	0.001	<0.001
	Moderate-COVID (n = 22)	4.60 (4.09–5.16)^*^	3.68 (3.08–4.40)^* a^	3.77 (3.24–4.38)^* a^	3.59 (3.01–4.29)^* a^			
	Severe-COVID (n = 18)	5.52 (4.93–6.18)^* #^	4.47 (3.85–5.20)^* a^	4.47 (3.87–5.17)^* a^	4.78 (4.03–5.67)^* #^			
**Objective fatigability analysis**								
TF (%)	Control (n = 30)	70.38 (67.08–73.84)				0.040	0.003	0.001
	Moderate-COVID (n = 22)	73.69 (68.85–78.88)	65.68 (61.01–70.70)^a^	70.12 (66.22–74.25)	70.80 (67.15–74.66)^b^			
	Severe-COVID (n = 18)	79.90 (73.56–86.80)^*^	74.28 (70.10–78.71)^#^	71.12 (66.65–75.88)^a^	76.97 (72.18–82.08)^* c^			
TF normalized by total-TTI	Control (n = 30)	0.49 (0.42–0.58)						
	Moderate-COVID (n = 22)	0.45 (0.36–0.55)	0.59 (0.49–0.72)^a^	0.50 (0.42–0.60)	0.48 (0.41–0.57)	0.61	0.52	0.40
	Severe-COVID (n = 18)	0.47 (0.34–0.64)	0.41 (0.34–0.50)^#^	0.56 (0.42–0.73)	0.38 (0.30–0.48)^c^			

Footnote: CI = confidence interval; n = number of participants; FSS = Fatigue severity scale; TF = Torque fatigability; TTI = Torque-time integral; a = different from baseline assessment intragroup; b = different from assessment 2 intragroup; c = different from assessment 3 intragroup; * = different from the Control assessment; # = different from Moderate-COVID at the same assessment. (p < 0.05). All outcomes were analyzed considering sex as a moderator (lowest QIC and p < 0.05 for sex).

### Assessment of functionality, muscle architecture and electrical neuromuscular abnormalities

Data regarding the 30s-STS (Table S1 in S1 File and Data S2 in S2 File) and SET (Table S2 in S1 File and Data S4 in S2 File) are available in the supporting information.

Regarding muscle architecture (Table 3 and Data S3 in S2 File), an interaction between group and assessment was observed in PA, specifically for the VM muscle (p = 0.002). Moderate-COVID patients exhibited higher PA values compared to the control group at baseline (p < 0.001) and in assessment 2 (p = 0.042), as well as compared to severe-COVID patients in assessment 4 (p = 0.015). Intragroup comparisons showed that assessment 3 of moderate-COVID patients displayed a lower PA compared to baseline (p = 0.002), while assessment 4 of severe-COVID patients showed a smaller PA compared to baseline (p = 0.005) and assessment 2 (p = 0.016).

**Table 3:** Muscle ultrasound comparisons for the knee extensors.

		Baseline assessment	Assessment 2	Assessment 3	Assessment 4	GEE (*p* values)		
	Groups	Mean (95% CI)	Mean (95% CI)	Mean (95% CI)	Mean (95% CI)	Group	Assessment	Group* Assessment
**Ultrasound analysis for rectus femoris**								
Pennation angle (°)	Control (n = 30)	12.72 (11.76–13.76)				0.792	0.243	0.264
	Moderate-COVID (n = 22)	13.09 (11.91–14.38)	12.33 (11.24–13.53)	11.78 (10.71–12.95)	11.98 (10.72–13.38)			
	Severe-COVID (n = 18)	12.40 (11.41–13.48)	12.78 (11.13–14.68)	13.40 (12.28–14.63)	11.40 (9.87–13.17)			
Fascicular length (cm)	Control (n = 30)	8.25 (7.50–9.08)				0.022	0.189	0.127
	Moderate-COVID (n = 22)	7.94 (7.05–8.94)	8.42 (7.48–9.48)	8.67 (8.00–9.41)	8.36 (7.62–9.17)			
	Severe-COVID (n = 18)	7.45 (6.88–8.05)	7.78 (6.64–9.11)	7.57 (6.84–8.38)	8.62 (7.75–9.58)			
Echogenicity (absolute values)	Control (n = 30)	44.20 (41.11–47.53)				<0.001	0.003	0.002
	Moderate-COVID (n = 22)	42.64 (38.86–46.80)^c^	42.75 (38.61–47.35)^c^	39.97 (36.30–44.00)	41.98 (38.23–46.09)^c^			
	Severe-COVID (n = 18)	55.54 (50.84–60.68)^* #^	53.93 (48.83–59.55)^* #^	52.33 (48.07–56.98)^* #^	50.69 (45.11–56.97)^a #^			
Muscle thickness (cm)	Control (n = 30)	1.73 (1.63–1.84)				0.066	0.048	0.050
	Moderate-COVID (n = 22)	1.76 (1.63–1.91)	1.87 (1.73–2.01)^a^	1.85 (1.72–2.00)^a^	1.85 (1.73–1.99)^a^			
	Severe-COVID (n = 18)	1.55 (1.40–1.72)^#^	1.59 (1.48–1.72)^#^	1.67 (1.53–1.83)	1.65 (1.51–1.81)^#^			
**Ultrasound analysis for vastus lateralis**								
Pennation angle (°)	Control (n = 30)	10.59 (9.45–11.87)				0.134	0.102	0.115
	Moderate-COVID (n = 22)	12.32 (10.97–13.82)	11.47 (10.20–12.89)	10.64 (9.54–11.86)	10.88 (9.40–12.60)			
	Severe-COVID (n = 18)	11.06 (9.71–12.59)	11.65 (10.25–13.24)	11.17 (10.07–12.39)	10.46 (9.51–11.51)			
Fascicular length (cm)	Control (n = 30)	10.87 (9.87–11.97)				0.002	<0.001	<0.001
	Moderate-COVID (n = 22)	7.52 (6.81–8.30)^*^	9.03 (8.16–9.99)^a *^	9.74 (8.90–10.65)^a^	9.34 (8.66–10.07)^a *^			
	Severe-COVID (n = 18)	8.55 (7.56–9.67)^*^	8.89 (7.93–9.95)^*^	9.45 (8.30–10.76)^a^	11.02 (9.57–12.70)^a b c^			
Echogenicity (absolute values)	Control (n = 30)	46.77 (44.28–49.40)				<0.001	0.625	0.254
	Moderate-COVID (n = 22)	48.30 (44.00–53.02)	48.58 (45.30–52.10)	48.64 (45.51–51.98)	49.19 (45.58–53.08)			
	Severe-COVID (n = 18)^**, ##^	61.10 (56.22–66.41)	58.86 (53.91–64.26)	58.23 (53.56–63.31)	56.47 (52.37–60.88)			
Muscle thickness (cm)	Control (n = 30)	1.85 (1.71–2.01)				0.427	<0.001	<0.001
	Moderate-COVID (n = 22)	1.57 (1.40–1.77)^*^	1.72 (1.56–1.89)^a^	1.78 (1.61–1.97)^a^	1.75 (1.58–1.94)^a^			
	Severe-COVID (n = 18)	1.55 (1.40–1.72)^*^	1.75 (1.54–1.99)^a^	1.74 (1.55–1.96)^a^	1.92 (1.72–2.14)^abc^			
**Ultrasound analysis for vastus medialis**								
Pennation angle (°)	Control (n = 30)	13.39 (12.32–14.55)				0.050	0.003	0.002
	Moderate-COVID (n = 22)	16.08 (15.10–17.12)^*^	15.15 (13.93–16.49)^*^	14.00 (12.98–15.10)^a^	14.89 (13.72–16.15)			
	Severe-COVID (n = 18)	14.84 (12.93–17.03)	14.78 (12.97–16.83)	13.62 (12.06–15.39)	12.21 (10.56–14.12)^a b #^			
Fascicular length (cm)	Control (n = 30)	8.04 (7.41–8.73)				0.303	<0.001	<0.001
	Moderate-COVID (n = 22)	6.70 (5.99–7.50)^*^	7.43 (6.68–8.27)^a^	7.86 (6.94–8.90)^a^	7.73 (7.07–8.45)^a^			
	Severe-COVID (n = 18)	6.06 (5.26–6.97)^*^	7.28 (6.36–8.34)^a^	7.30 (6.26–8.50)	9.14 (7.83–10.66)^a b c^			
Echogenicity (absolute values)	Control (n = 30)	42.16 (39.79–44.66)				<0.001	0.008	0.025
	Moderate-COVID (n = 22)	43.67 (39.87–47.84)	45.48 (42.39–48.79)	41.79 (38.13–45.80)^b^	42.32 (38.73–46.23)^b^			
	Severe-COVID (n = 18)	59.11 (53.30–65.57)^* #^	57.11 (52.10–62.60)^* #^	54.83 (51.20–58.72)^a * #^	55.14 (51.84–58.66)^* #^			
Muscle thickness (cm)	Control (n = 30)	1.85 (1.70–2.00)				0.226	<0.001	<0.001
	Moderate-COVID (n = 22)	1.79 (1.63–1.96)	1.89 (1.72–2.09)	1.85 (1.67–2.06)	1.95 (1.78–2.13)^a^			
	Severe-COVID (n = 18)	1.50 (1.34–1.68)^* #^	1.79 (1.65–1.94)^a^	1.68 (1.47–1.92)	1.84 (1.63–2.06)^a^			
Knee extensor muscle volume (cm)	Control (n = 30)	774.21 (640.48–935.85)				0.009	0.008	<0.001
	Moderate-COVID (n = 22)	748.39 (597.81–936.91)	783.29 (624.12–983.05)	792.60 (642.55–977.69)^a^	834.33 (656.87–1059.73)^a^			
	Severe-COVID (n = 18)	606.94 (474.10–777.00)	735.62 (599.36–902.86)^a^	675.51 (544.61–837.87)^ab^	734.02 (594.91–905.66)^ac^			

Footnote: CI= confidence interval; n= number of participants; RF= rectus femoris; VL= Vastus lateralis; VM= Vastus medialis; a= different from baseline assessment; b= different from assessment 2; c= different from assessment 3; #= different from moderate-COVID at the same assessment; *= different from the Control; ##= different from moderate-COVID, main effect of group; **= different from control, main effect of group. (p<0.05).

All outcomes were analyzed considering sex as a moderator (lowest QIC and p < 0.05 for sex), except for RF fascicular length, VL pennation angle, VL muscle thickness and knee extensor muscle volume which were analyzed using age as a moderator (lowest QIC and p < 0.05 for age). VL fascicular length and VM fascicular length were analyzed without a moderator (lowest QIC).

For the FL analysis, in the VL muscle, an interaction between group and assessment (p < 0.001) showed that both COVID groups presented shorter FL values compared to the control group, which was particularly evident at baseline (severe-COVID vs control: p = 0.002; moderate-COVID vs control: p < 0.001) and assessment 2 (severe-COVID vs control: p = 0.007; moderate-COVID vs control: p = 0.009) for both groups, and in assessment 4 for moderate-COVID (p = 0.017). Intragroup comparisons indicated shorter FL values at baseline for COVID groups (p < 0.05). Similar patterns were observed in the VM muscle, where an interaction between group and assessment (p < 0.001) revealed that the COVID groups exhibited shorter FL values compared to the control group at baseline (severe-COVID vs control: p < 0.001; moderate-COVID vs control: p = 0.009). Regarding the intragroup comparisons, moderate-COVID showed shorter FL values at baseline compared to subsequent assessments (p < 0.05). Severe-COVID patients exhibited shorter FL values at baseline compared to assessments 2 (p = 0.014) and 4 (p < 0.001), with assessment 4 showing greater FL values compared to assessments 2 (p = 0.001) and 3 (p = 0.033). For the RF muscle, there was a group effect (p = 0.022); however, no differences were observed in the post hoc analyses.

For echogenicity analysis, an interaction between group and assessment was observed for the RF muscle (p = 0.002) and VM muscle (p = 0.025). Severe-COVID patients exhibited higher echogenicity values compared to the other groups in most assessments (p < 0.05). Regarding intragroup comparisons in the RF muscle, moderate-COVID patients showed lower echogenicity in assessment 3 (p < 0.05) compared to the other assessments, while severe-COVID patients displayed lower values in assessment 4 compared to baseline (p = 0.005). In the VM muscle, the intragroup analysis showed that moderate-COVID patients demonstrated higher echogenicity in assessment 2 compared to assessments 3 (p = 0.002) and 4 (p = 0.023), and at baseline the severe-COVID patients exhibited higher echogenicity compared to assessment 3 (p = 0.047). For the VL muscle, only a group interaction was observed (p < 0.001), indicating that severe-COVID patients presented greater echogenicity compared to the moderate-COVID (p < 0.001) and control groups (p < 0.001).

Regarding the MT analysis, an interaction between group and assessment was observed for the RF muscle (p = 0.05). Severe-COVID patients presented lower MT values in the RF muscle compared to moderate-COVID patients at baseline (p = 0.05), and in assessments 2 (p = 0.004) and 4 (p = 0.05). In the intragroup comparisons, moderate-COVID patients exhibited lower MT at baseline compared to the other assessments (p < 0.05). For the VL muscle, an interaction between group and assessment was observed (p < 0.001). The severe-COVID and moderate-COVID groups presented lower MT values in the VL muscle compared to the control group at baseline (p = 0.007 and 0.025, respectively). In the intragroup comparisons, moderate-COVID patients exhibited lower MT at baseline compared to the other assessments (p < 0.05). A similar pattern was observed in the severe group (p < 0.05), which also showed higher MT values at the fourth assessment compared to the other time points (p < 0.05). Regarding the VM muscle, severe-COVID patients exhibited lower MT values compared to moderate-COVID (p = 0.018) and control groups (p = 0.003) at baseline. In the intragroup comparisons, severe-COVID patients had lower MT at baseline compared to assessments 2 (p < 0.001) and 4 (p = 0.002), while moderate-COVID patients showed lower baseline MT compared to assessment 4 (p = 0.027).

Regarding the MV analysis, an interaction between group and assessment was observed (p < 0.001), only intragroup differences were found. The moderate-COVID group showed higher MV at assessments 3 (p = 0.04) and 4 (p = 0.013) compared to baseline. The severe-COVID group also presented a lower MV at baseline compared to the other assessments (p < 0.05). Additionally, MV at assessment 3 was lower than at assessments 2 (p = 0.018) and 4 (p = 0.034).

### Torque, torque-time integral, electromyography activity, and rate of force development

**Fig 2:**
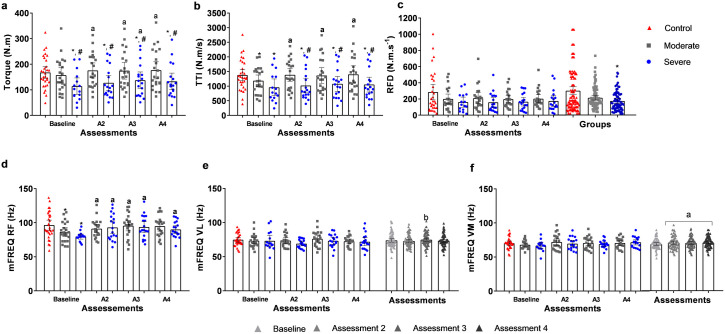
Torque, torque-time integral (TTI), electromyography activity and rate force of development (RFD) of the three groups in the assessments obtained during the two initial maximal voluntary contractions. (a) Torque maximal. (b) TTI. (c) RFD; (d) Median frequency (mFREQ) of RF, mFREQ of VL, and mFREQ of VM. A2 = assessment 2; A3 = Assessment 3; A4 = assessment 4; a = different from baseline; b = different from assessment 3; * = different from control group; # = different from moderate-COVID. (p < 0.05).

The means of data obtained from the two initial MVCs for the variables torque, TTI, electromyographic activity, and RFD are presented in Fig 2, Table 4, and Supplementary Data S5 in S2 File. A significant interaction was found between group and assessment for torque (p = 0.012) and TTI (p = 0.04), with the severe-COVID exhibiting lower values than the other groups in most assessments (p < 0.05) (Fig 2, Table 3 and Data S5 in S2 File). The moderate-COVID group also showed a lower TTI than the control at baseline (p = 0.007). In intragroup comparisons, moderate-COVID had lower values for both outcomes at baseline (p < 0.05), while severe-COVID presented lower torque at baseline compared to assessment 3 (p = 0.047).

**Table 4:** Torque, torque-time integral (TTI), rate force of development (RFD), and electromyography activity comparisons for the knee extensors obtained during the two initial maximal voluntary contractions.

		Baseline assessment	Assessment 2	Assessment 3	Assessment 4	GEE (*p* values)		
	Groups	Mean (95% CI)	Mean (95% CI)	Mean (95% CI)	Mean (95% CI)	Group	Assessment	Group* Assessment
**Isometric Knee-Extension torque production**								
Torque (Nm)	Control (n = 30)	168.22 (154.59–183.05)				<0.001	0.018	0.012
	Moderate-COVID (n = 22)	152.56 (138.58–167.95)	172.89 (156.90–190.52)^a^	170.67 (154.03–189.11)^a^	171.13 (154.62–189.40)^a^			
	Severe-COVID (n = 18)	114.02 (96.18–135.17)^* #^	124.88 (108.49–143.74)^* #^	134.56 (116.06–156.00)^a * #^	129.23 (112.59–148.33)^* #^			
TTI (Nm.s)	Control (n = 30)	1382.23 (1265.86–1509.30)				0.001	0.084	0.004
	Moderate-COVID (n = 22)	1162.36 (1066.19–1267.19)^*^	1369.58 (1237.66–1515.55)^a^	1331.80 (1191.71–1488.36)^a^	1355.48 (1213.95–1513.51)^a^			
	Severe-COVID (n = 18)	958.59 (788.78–1164.97)^*^	1002.54 (863.40–1164.10)^* #^	1057.28 (907.73–1231.46)^* #^	1025.37 (896.32–1173.00)^* #^			
RFD (N.m.s^-1^)	Control (n = 30)	260.98 (203.18–335.22)				0.024	0.996	0.904
	Moderate-COVID (n = 22)	204.63 (155.75–268.86)	229.89 (157.03–336.56)	207.62 (163.04–264.39)	206.59 (169.18–252.27)			
	Severe-COVID (n = 18)^**^	175.28 (119.47–257.17)	153.06 (118.17–198.26)	165.31 (124.74–219.06)	171.90 (133.15–221.93)			
**Electromyography activity**								
RF mFREQ (Hz)	Control (n = 30)	96.94 (90.45–103.90)				0.15	<0.001	<0.001
	Moderate-COVID (n = 22)	86.81 (81.03–93.00)^*^	91.64 (86.41–97.19)	95.79 (89.17–102.90)^a^	95.43 (89.12–102.17)^a^			
	Severe-COVID (n = 18)	80.51 (76.86–84.34)^*^	93.17 (85.99–100.94)^a^	94.16 (87.01–101.89)^a^	90.43 (85.50–95.65)^a^			
VL mFREQ (Hz)^e^	Control (n = 30)	74.81 (71.64–78.13)				0.46	0.04	0.055
	Moderate-COVID (n = 22)	73.31 (69.44–77.41)	73.55 (69.73–77.58)	76.16 (71.79–80.81)	72.62 (69.66–75.71)			
	Severe-COVID (n = 18)	73.30 (66.67–80.60)	69.10 (66.33–71.99)	73.24 (68.95–77.80)	71.93 (66.82–77.44)			
VM mFREQ (Hz)^b,c,d^	Control (n = 30)	70.31 (67.38–73.37)				0.800	0.047	0.176
	Moderate-COVID (n = 22)	67.96 (65.66–70.35)	72.04 (67.60–76.78)	70.53 (66.92–74.34)	70.56 (67.45–73.80)			
	Severe-COVID (n = 18)	66.69 (62.88–70.73)	69.59 (65.57–73.85)	68.89 (66.28–71.61)	71.56 (68.18–75.10)			

Footnote: CI= confidence interval; n= number of participants; RF= Rectus femoris; VL= Vastus lateralis; VM= Vastus medialis; TTI= torque-time integral; RFD= rate of force development; mFREQ= medium frequency; a= different from baseline assessment; d= assessment 2 different from baseline, main effect of assessment; c= assessment 3 different from baseline, main effect of assessment; d= assessment 4 different from baseline, main effect of assessment; e= assessment 3 different from assessment 2, main effect of assessment; #= different from moderate-COVID at the same assessment; *= different from the Control. **= different from control, main effect of group (p<0.05).

All outcomes were analyzed considering sex as a moderator (lowest QIC and p < 0.05 for sex), except for VL mFREQ, and VM mFREQ, which were analyzed without a moderator (lowest QIC).

For the RF mFREQ, a significant interaction between group and assessment (p < 0.001) showed that both COVID groups exhibited lower values than the control at baseline (p < 0.05). In intragroup comparisons, severe-COVID demonstrated a lower mFREQ at baseline compared to assessments 2 (p = 0.001), 3 (p < 0.001), and 4 (p < 0.001), while moderate-COVID had lower values at baseline compared to assessments 3 (p = 0.003) and 4 (p = 0.004). For the VL (p = 0.040) and VM (p = 0.047) muscles, significant effects of assessment were observed. The VL showed a lower value in assessment 2 compared to assessment 3 (p = 0.006), while the VM had lower values at baseline compared to the other assessments (p < 0.05). Regarding RFD, the group effect (p = 0.024) indicated that severe-COVID had a lower value than the control (p = 0.012).

### Objective fatigability assessments

The significant interaction observed between group and assessment for TF (p = 0.001) (Table 2 and Data S6 in S2 File) demonstrated that severe-COVID exhibited higher values than moderate-COVID in assessment 2 (p = 0.009) and compared to moderate-COVID and the control at baseline (p = 0.012) and assessment 4 (p = 0.031). In the intragroup comparisons, moderate-COVID had lower TF in assessment 2 compared to baseline (p = 0.006) and assessment 4 (p = 0.045). For severe-COVID, assessment 3 had lower values than baseline (p = 0.012) and assessment 4 (p = 0.004).

**Fig 3:**
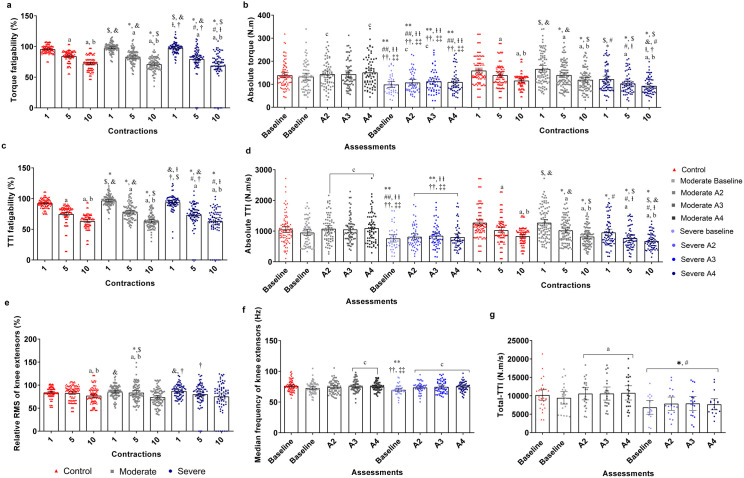
Torque, torque-time integral (TTI) and electromyography activity analysis of knee extensors during the fatigue protocol. (a) Torque fatigability, (b) Absolute torque. (c) TTI fatigability, (d) Absolute TTI. (e) Relative root mean squared (RMS) of knee extensors; (f) Median frequency of knee extensors; (g) Total-TTI. A2 = assessment 2; A3 = Assessment 3; A4 = assessment 4; a = different from contraction 1 intragroup; b = different from contraction 5 intragroup; c = different from baseline assessment intragroup; d = different from assessment 2 intragroup; e = different from assessment 3 intragroup; * = different from contraction 1 of the Control; $ = different from contraction 5 of the Control; & = different from contraction 10 of the Control; # = different from contraction 1 of moderate-COVID; ⱡ = different from contraction 5 of moderate-COVID; † = different from contraction 10 of moderate-COVID; ** = different from the Control assessments; ## = different from baseline assessment of moderate-COVID; ⱡ ⱡ = different from assessment 2 of moderate-COVID; †† = different from assessment 3 of moderate-COVID; ‡‡ = different from assessment 4 of moderate-COVID. (p < 0.05).

A significant interaction between group and total-TTI assessment (p = 0.017) (Fig 3, Table S4 in S1 File, and Data S6 in S2 File) revealed that severe-COVID exhibited the lowest total-TTI compared to moderate-COVID and control in all assessments (p < 0.05). In the intragroup comparisons, moderate-COVID displayed the lowest value at baseline compared to other assessments (p < 0.05).

For the fatigue protocol, a significant interaction between group and contractions for TF (p = 0.035) and TTI-F (p = 0.013) revealed that all groups exhibited their highest values at MVC 1 compared to MVCs 5 and 10, with MVC 10 showing the lowest value compared to MVC 5 (p < 0.05) for all groups. Some trivial differences were also found between groups and contractions (p < 0.05) (Fig 3, Table S4 in S1 File and Data S7 in S2 File).

For absolute maximal torque (p = 0.026) and TTI (p = 0.006) (Fig 3, Table S4 in S1 File and Data S7 in S2 File), an interaction between group and assessment revealed that severe-COVID had the lowest values compared to the other groups in most assessments (p < 0.05). In the intragroup comparisons, the baseline for moderate-COVID had lower absolute maximal torque than assessments 2 (p = 0.031) and 4 (p = 0.004). For severe-COVID, the baseline showed lower values than assessments 2 (p = 0.046) and 3 (p = 0.016). Intragroup comparisons for absolute TTI indicated that moderate-COVID had the lowest value at baseline compared to assessments 2 (p = 0.001), 3 (p = 0.002), and 4 (p = 0.003). An interaction between group and contraction demonstrated that severe-COVID exhibited the lowest absolute maximal torque (p = 0.020) and TTI (p = 0.014) in most between-group comparisons during all MVCs (p < 0.05).

An interaction between group and contraction (p = 0.037) showed that moderate-COVID and control had higher relative RMS values in MVCs 1 and 5 compared to MVC 10 (p < 0.05). For mFREQ, an interaction between group and assessment (p = 0.002) revealed that the control (p = 0.001) and assessments 3 (p = 0.006) and 4 (p = 0.004) of moderate-COVID had higher mFREQ compared to severe-COVID at baseline. Intragroup comparisons indicated that both COVID‑19 groups had lower mFREQ at baseline compared to most assessments (p < 0.05) (Fig 3, Table S4 in S1 File, and Data S7 in S2 File). Information regarding PE is available in Table S4 in S1 File and Data S8 in S2 File.

## Discussion

The results of the current study showed that severe-COVID participants exhibited greater perceived fatigue, worse functional performance, changes in muscle architecture, altered neuromuscular function, and significantly lower torque, TTI, and total-TTI, which may be related to reduced work capacity and influence fatigability, especially perceived fatigability. Conversely, moderate-COVID participants showed lower TTI than the control only in the initial assessment, while demonstrating improvements in torque, TTI, and total-TTI over the following year. These results reflect not only the intrinsic characteristics of COVID‑19 but also suggest that the clinical profile of the participants may have influenced the study findings. Individuals in the severe COVID‑19 group exhibited risk factors frequently associated with worse disease outcomes [39], such as older age, higher body weight and BMI, a greater prevalence of comorbidities, and lower levels of physical activity, which may have contributed to a more compromised baseline status [39–41]. These factors are widely recognized as predictors of increased disease severity and, collectively, can negatively impact the musculoskeletal system, thereby increasing patient vulnerability [42,43]. Furthermore, severe-COVID patients often require greater care, including hospitalization, which results in bed rest [39,40]. Our participants in the severe-COVID group spent an average of 21 days in the intensive care unit (ICU), and many of them were already sedentary, which likely affected their physical-functional capacity. Hospitalization during the acute phase of COVID‑19 is associated with a higher risk of re-hospitalization, long-term sequelae, and an increased risk of death, that can persist for up to three years [4,10]. Moreover, a sedentary lifestyle increases the risk of premature death [44] and has negative effects on the musculoskeletal system [45]. Nevertheless, these characteristics are part of the clinical picture typically associated with severe COVID‑19 [39–41]. Although some alterations in neuromuscular function were observed, objective data indicate that the capacity to maintain force relative to maximal capacity was preserved across groups, suggesting that fatigability is not significantly impaired. Thus, although these factors may have influenced our results, the findings also reflect longitudinal changes in individuals with different levels of disease severity, providing relevant insights into the systemic effects of COVID‑19, the significant reductions in muscle activity, structural limitations that may contribute to reduced muscle performance, and the recovery process of these patients over time.

Participants in the COVID‑19 groups reported significantly higher perceived fatigue than controls. Severe-COVID participants consistently exhibited significant higher perceived fatigue (≥4) across all assessments, whereas those with moderate COVID showed significant higher perceived fatigue only at baseline. This aligns with existing literature indicating that even individuals with moderate disease can experience persistent fatigue [3,10,46,47]. Although the groups differed in baseline health characteristics, participants in the severe-COVID group continued to report significant perceived fatigue and exhibit greater fatigability one year after infection. This suggests that recovery may be slower or incomplete in this group, potentially impacting functionality [10]. Severe-COVID participants consistently received scores below normal limits in the functionality test, for both sexes at baseline and below the male threshold in subsequent assessments [48], indicating compromised functionality, likely attributed to hospitalization-related disuse or reduced use. Prolonged inactivity can lead to muscle mass loss, impairing muscle function and impacting independence and quality of life [49–51]. The strength of the knee extensors is linked to functional activities, which demand a rapid and precise combination of force and speed, especially in older adults [52–54]. Although our study included various age groups, strength, explosive strength, and work capacity, were compromised in severe-COVID. This fact is important, as approximately 32% of individuals with long COVID do not return to work due to diminished physical condition, even one year after onset [3].

Ultrasound analysis also revealed distinct features in severe-COVID participants, especially at baseline. These participants showed higher echogenicity and lower MT, indicating reduced muscle quality. Similar patterns have previously been documented in ICU patients [49,50,55], suggesting that alterations resulting from hospitalization and prolonged bed rest may persist beyond the acute phase, characterizing a post-COVID myopathy phenotype. Increased echogenicity could suggest infiltration of adipose and connective tissue and increased muscle necrosis and chronic inflammatory processes, all of which contribute to deteriorated muscle quality by compromising muscle architecture and contractile capacity [12,13,50]. Echogenicity has also been linked to functional performance [12], with higher echogenicity serving as a strong indicator of poorer functional prognosis [50], which may explain the impairments observed in the severe-COVID group. Furthermore, greater MT can lead to increased muscle strength due to a larger cross-sectional area and greater physiological adaptations, such as enhanced recruitment of motor units and improved neuromuscular efficiency [21,49,56]. Significant differences in FL were also observed between groups, with the severe-COVID group showing lower values, particularly at baseline. These findings support the notion of altered muscle architecture, likely due to reduced muscle mass. Shorter fascicles may have important functional implications, as they are associated with decreased shortening capacity and contraction speed, ultimately impairing performance in tasks that require strength, power, and mobility [57]. Additionally, these changes in muscle architecture negatively affect mechanical efficiency, promoting early onset of fatigue and increasing the risk of musculoskeletal injuries [49]. Thus, patients who experience substantial loss of muscle cross-sectional area during hospitalization may not recover even six months after discharge [58]. Muscle mass loss can vloss can lead to higher rates of fatigue and myalgia, potentially explaining our findings [58].

Prolonged disuse, combined with disease, medications, and other interventions in hospital settings, can affect neuromuscular tissues [59,60]. Electrical neuromuscular abnormalities are observed in hospitalized patients [59–61] and in patients hospitalized for COVID‑19 [61]. Long COVID individuals also present myopathic changes, with electromyographic abnormalities linked to damage in the muscles, terminal nerves, and motor endplate [62]. Furthermore, changes in electrical neuromuscular function appear to be related to the risk of long COVID muscle fatigue [63]. Indeed, we demonstrated that participants with severe COVID exhibit lower values of mFREQ, as well as changes in muscle architecture, which may have contributed to performing less total work. Additionally, severe-COVID participants exhibited higher values in most SET outcomes, particularly chronaxie, for all analyzed muscles. Higher chronaxie (≥1000 µs) may indicate peripheral electrical neuromuscular abnormalities [24,64]. While the average chronaxie value was normal, 22.22% of severe-COVID participants exhibited abnormalities in the RF muscle, and 16.66% showed abnormalities in the VM muscle at baseline. This underscores the need for comprehensive evaluations, as these factors can influence rehabilitation programs and recovery rates post-discharge [24,65,66].

Perception of fatigue may be related to decreased contractile strength in muscle fibers, leading to reduced contraction mechanisms [2,67]. Herein, we analyzed TF, which represents the decline in torque output, where lower values indicate less total work and TF normalized by total-TTI as objective measures of fatigue. Interestingly, in these objective measures of fatigue, the severe-COVID group showed significantly higher TF values at baseline and during assessments 2 and 4, and no significant differences for TF normalized by total-TTI, suggesting important group differences. Our data corroborate the findings of Fietsam et al. [29] who assessed fatigue both subjectively, using a scale, and objectively, with indices derived from torque and work fatigue calculated from an isokinetic fatigue task [29]. Although individuals with long COVID reported greater perceived fatigue compared to those without the condition, no differences were found in objective fatigability [29]. A potential explanation for this discrepancy is the multifaceted nature of fatigue, shaped by subjective elements, which permit psychosomatic factors to impact the perception of fatigue [2,29,68]. These changes may cause the body to conserve energy and maintain steady basal activity during fatiguing tasks, rather than pushing to maximum capacity to avoid excessive exhaustion [68].

All groups showed a reduction in absolute torque and TTI during the fatigue protocol, with only trivial differences between groups for other outcomes (TF, TTI-F, and PE). However, severe-COVID presented lower total mFREQ at baseline compared to controls. During fatigue, the mFREQ signal decreases, likely due to changes in action potential characteristics, firing rates, additional motor unit recruitment, and effort levels [69,70]. However, caution is necessary when interpreting electromyography results, as factors like adipose tissue can influence measurements [69]. While this finding may not be highly significant, it helps explain the lower torque, TTI, and total-TTI consistently observed in the severe-COVID. Unlike moderate-COVID participants, who showed an increase in TTI over time, severe-COVID participants did not demonstrate a statistically significant improvement. This indicates more pronounced differences related to work capacity, as total-TTI reflects the intensity and duration of contractions, suggesting a reduced capacity for isometric work over time and a greater tendency toward fatigability [31]. Research indicates that individuals with long COVID often experience reduced exercise capacity and altered muscle metabolism, shifting toward a less oxidative muscle fiber phenotype [7,11,71]. Although we did not directly assess muscle metabolism, the severe-COVID group showed more pronounced changes, potentially contributing to reduced torque production and TTI. However, it is important to highlight that although severe-COVID participants performed less total work during the tests, this likely reflects reduced muscle mass and structural limitations rather than impaired fatigue resistance. The diminished mechanical output seems to be due to these structural and architectural constraints rather than a true deficit in fatigue resistance. This distinction is supported by the preserved TF across groups, indicating that the capacity to sustain force relative to maximal strength was not disproportionately affected in the severe-COVID group. The mechanisms underlying the persistent fatigue of long COVID seem to be a complex phenomenon, influenced by neuropsychological, peripheral, and central mechanisms. Peripheral factors include impaired nerve impulse conduction, neuromuscular junction dysfunction, and changes in muscle contractile properties [6,47,72,73]. Muscles appear to undergo changes during recovery, some of which are linked to the acute infection [16]. Meanwhile, central factors involve changes in spinal and supraspinal mechanisms, including alterations in neurotransmitter levels, neuronal excitability, inflammation, and demyelination [6,47,72]. Among the central factors, structural brain changes in the thalamus, basal ganglia [74], and in microvascular structures on the white matter [75] also appear to be associated with fatigue in long COVID and correlate with the severity of fatigue and other symptoms, such as short-term memory problems [74]. Additionally, COVID‑19 individuals with neurological manifestations often exhibit fatigue and deficits in executive functions, potentially linked to a hyperinflammatory state caused by cortical impairment of GABAergic neurotransmission [47]. Although our study did not analyze central factors of fatigue, they likely play a significant role.

Our study has limitations. Firstly, the inability to determine the ideal sample size means the participants may not fully represent the diversity of COVID‑19 presentations and recovery trajectories. Secondly, reliance on perceived measures may introduce bias due to individual variations in interpretation. However, we also used objective measures of fatigability and evaluated contributing factors, offering valuable insights into fatigue. We also faced some instrumental limitations that hindered more comprehensive analysis of the data, including the assessment of certain outcomes such as muscle cross-sectional area, which could not be collected due to the lack of appropriate equipment and which could have offered a more in-depth interpretation of the torque and fatigability results. Another relevant aspect to be considered is that the social restrictions imposed by the pandemic substantially impacted the recruitment and assessment of the control group. Due to the high rate of COVID‑19 infection during the follow-up period, it was necessary to limit data collection from these participants to a single assessment. This limitation compromised the matching between the COVID‑19 groups and the control group with respect to baseline characteristics such as age, activity level, and BMI, potentially introducing confounding factors that may have influenced the results of the present study. Another important limitation was the lack of control for pre-existing conditions such as chronic fatigue syndrome, myalgic encephalomyelitis, or fibromyalgia. These conditions are associated with reduced neuromuscular and cardiorespiratory fitness and may be present among individuals with long COVID, potentially having a negative impact on performance [76]. The possible presence of these conditions should be considered when planning future research. Additionally, monitoring over a one-year period presented challenges, as factors like new viral infections and health treatments could not be controlled. The persistence of fatigue indicators over a year differentiates the severe group from the moderate group. However, both groups shared baseline characteristics considering the COVID‑19 classification and known risk factors for greater infection severity of the disease, such as higher comorbidities, age, body weight, and the period of disuse [39]. These shared factors may confound the influence of Long COVID symptoms and should be considered for in future studies.

## Conclusion

Participants with severe COVID‑19 demonstrated higher levels of perceived fatigue, reduced functional performance, and greater impairments in muscle architecture and neuromuscular function, contributing to increased fatigue perception and workload over the one-year follow-up. In contrast, moderate COVID‑19 participants had more pronounced impairments in muscle architecture and neuromuscular function, in addition to reduced torque and TTI at baseline. These findings highlight the role of musculoskeletal and neural mechanisms in post-infection fatigue, emphasizing the importance of targeted, mechanism-based rehabilitation strategies during recovery.

## Supporting information

S1 File Supplementary results. Supplementary results and tables S1 to S4.
(DOCX)

S2 File Supplementary data. Supplementary data tables S1 to S8.
(XLSX)

S3 File STROBE Checklist. Completed STROBE cohort checklist.
(DOCX)
