# Supplementary material for: Fatigue and neuromuscular function in long COVID: A one-year follow-up study
Source: PLoS One. 2025 Sep 24;20(9):e0332242. doi: 10.1371/journal.pone.0332242 (PMC12459844; doi:10.1371/journal.pone.0332242)
Supplement: S1 File — Supplementary results and tables S1 to S4. (DOCX) [file pone.0332242.s001.docx]

Supporting information 1. Supplementary results – Supplementary results and tables S1 to S4

Manuscript: **FATIGUE AND NEUROMUSCULAR FUNCTION IN LONG-COVID: A ONE-YEAR FOLLOW-UP STUDY**

**RESULTS**

**Functionality assessment**

In the 30-second sit-to-stand test (Table S1 and data S2 in the supporting information), there was a significant interaction between group and assessment (p<0.001). Severe-COVID patients achieved fewer repetitions than the other groups across all assessments (p<0.05). Additionally, the COVID groups showed fewer repetitions at baseline compared to the other assessments (p <0.05).

**Electrical neuromuscular abnormalities assessment**

The results for the stimulus electrodiagnostic test assessment can be seen in table S2 and data S4 in the supporting information. Significant interactions between group and assessment were observed for rheobase in the RF (p<0.001), VL (p=0.011), and VM (p<0.001) muscles. Severe-COVID patients exhibited higher rheobase values compared to moderate-COVID and the control group across these muscles in most assessments (p<0.05). Specifically, in the RF analysis, moderate-COVID showed higher values compared to control at baseline (p=0.028). In the intragroup comparisons for RF rheobase, moderate-COVID presented the lowest value at baseline compared to assessments 3 (p<0.05) and 4 (p<0.05), and assessment 2 had a lower value compared to assessment 4 (p<0.05). For VL rheobase, baseline values were lower compared to assessment 4 (p=0.022). In the VM rheobase intragroup comparisons, assessment 4 of moderate-COVID showed the highest value compared to baseline and assessments 2 (p<0.001) and 3 (p<0.001), while severe-COVID showed the highest value in assessment 4 compared to assessments 2 (p=0.041) and 3 (p=0.005).

For chronaxie, significant interactions between group and assessment were observed for RF (p<0.001), VL (p=0.002), and VM (p=0.005) muscles. Severe-COVID patients demonstrated higher chronaxie values compared to control in most assessments across all analyzed muscles (p<0.05). Moderate-COVID also showed higher values compared to control in assessment 2 in RF (p=0.048) and assessment 4 in VM (p=0.007). Additionally, in the VL muscle analysis, moderate-COVID displayed higher values compared to control at baseline (p=0.017) and in assessments 2 (p=0.001) and 3 (p=0.002). In severe-COVID, baseline values were higher in RF and VL muscle analyses compared to assessment 4 (p=0.036, p=0.012, p=0.006, respectively). In the VM muscle analysis, assessment 3 of moderate-COVID showed higher values compared to baseline (p<0.040) and assessment 4 (p<0.005).

Regarding the accommodation index (AI), significant interactions between group and assessment were observed for RF (p=0.015) and VM (p<0.001) muscles. Severe-COVID patients had higher accommodation values compared to moderate-COVID and control in most assessments for RF and VM muscles (p<0.05). In the intragroup comparisons for RF and VM muscles, assessment 4 showed higher values compared to the other assessments in both COVID groups (p<0.05). Furthermore, the analysis of VL muscle accommodation indicated a significant effect of group (p=0.031) and assessment (p=0.015), where severe-COVID patients exhibited higher values compared to moderate-COVID (p=0.036) and control (p=0.019). Assessment 4 also showed higher values compared to baseline (p=0.05) and assessment 3 (p=0.05).

For AI of the RF muscle, significant interactions between group and assessment (p<0.001) revealed lower values for severe-COVID patients compared to control at baseline (p=0.001) and assessment 3 (p=0.028) and compared to moderate-COVID at baseline (p=0.001). Moderate-COVID patients had higher AI values compared to control at baseline (p=0.001). In the intragroup comparisons for moderate-COVID, baseline values were higher compared to other assessments (p<0.05), while severe-COVID patients showed lower baseline values compared to assessments 2 (p=0.004) and 3 (p=0.044). In the VL muscle, an interaction between group and assessment (p=0.007) indicated that moderate-COVID patients presented higher values at baseline compared to assessments 3 (p=0.004) and 4 (p=0.024). Assessment 3 also showed the lowest AI values compared to assessment 2 (p=0.047). No significant differences were observed for VM muscle (p=0.649).

**Objective fatigability assessments, perceived exertion (PE)**

Regarding PE (table S4 and data S8 in the supporting information), a significant interaction between group, assessment, and contraction revealed that severe-COVID exhibited the highest PE before starting the fatigue protocol compared with the control group (p=0.003) at baseline. Additionally, for some MVCs at baseline, and assessments 3 and 4, a higher PE was observed for moderate-COVID compared with severe-COVID and the control groups (p<0.05). In intragroup comparisons, showed that values before the fatigue protocol were lower than at MVCs 5 and 10, with MVC 10 being higher than MVC 5 (p<0.05) across all groups. Additionally, the baseline displayed higher PE for severe-COVID (p<0.05) compared with the other assessments for most of the moments analyzed. For moderate-COVID, the PE before starting the fatigue protocol was lower in assessment 4 than baseline (p=0.006) and assessment 2 (p=0.028). In contraction 5, moderate-COVID also presented a lower PE in assessment 4 than baseline (p=0.041).

**Supplementary tables**

**Table S1. 30-second sit-to-stand test (30s-STS) comparisons.**

|  |  | **Baseline assessment** | **Assessment 2** | **Assessment 3** | **Assessment 4** | **GEE (*p* values)** | | |
| --- | --- | --- | --- | --- | --- | --- | --- | --- |
|  | **Groups** | **Mean (95% CI)** | **Mean (95% CI)** | **Mean (95% CI)** | **Mean (95% CI)** | **Group** | **Assessment** | **Group* Assessment** |
| 30s-STS | Control (n=30) | 15.93 (14.20 – 17.66) |  |  |  | <0.001 | <0.001 | <0.001 |
|  | Moderate-COVID (n=22) | 15.80 (14.03 – 17.57) | 18.08 (15.68 – 20.48)^a^ | 18.65 (16.15 – 21.16)^a^ | 18.51 (15.98 – 21.05)^a^ |  |  |  |
|  | Severe-COVID (n=18) | 10.10 (7.89 – 12.31)^* #^ | 12.31 (10.13 – 13.90)^* # a^ | 11.86 (10.05 – 13.72)^* # a^ | 12.74 (10.94 – 14.55)^* # a^ |  |  |  |
| Footnote: CI= confidence interval; n= number of participants; a= different from baseline assessment; #= different from moderate-COVID at the same assessment; *= different from the Control. (p<0.05). Analyzed considering age as a moderator (lowest QIC and p < 0.05 for age). | | | | | | | | |

**Table S2. Stimulus electrodiagnostic test (SET) analysis comparisons for the knee extensors**

|  |  | **Baseline assessment** | **Assessment 2** | **Assessment 3** | **Assessment 4** | **GEE (*p* values)** | | |
| --- | --- | --- | --- | --- | --- | --- | --- | --- |
|  | **Groups** | **Mean (95% CI)** | **Mean (95% CI)** | **Mean (95% CI)** | **Mean (95% CI)** | **Group** | **Assessment** | **Group* Assessment** |
| **SET for rectus femoris** | | | | |  |  |  |  |
| Rheobase | Control (n=30) | 9.95 (8.91 – 11.12) |  |  |  | <0.001 | <0.001 | <0.001 |
|  | Moderate-COVID (n=22) | 8.14 (7.03 – 9.42)^*^ | 8.51 (7.49 – 9.67) | 9.52 (8.47 – 10.71)^a^ | 10.61 (9.58 – 11.75)^a b^ |  |  |  |
|  | Severe-COVID (n=18) | 13.05 (11.79 – 14.45)^* #^ | 13.05 (11.67 – 14.58)^* #^ | 12.99 (11.67 – 14.47)^* #^ | 13.96 (12.75 – 15.29)^* #^ |  |  |  |
| Chronaxie (µs) | Control (n=30) | 175.41 (142.35 – 216.15) |  |  |  | 0.001 | <0.001 | <0.001 |
|  | Moderate-COVID (n=22) | 270.85 (188.01 – 390.20) | 245.69 (192.30 – 313.90)^*^ | 214.27 (175.87 – 261.05) | 202.62 (152.54 – 269.14) |  |  |  |
|  | Severe-COVID (n=18) | 644.97 (333.26 – 1248.23)^*^ | 307.24 (236.97 – 398.34)^*^ | 289.31 (220.66 – 379.31)^*^ | 322.18 (197.30 – 526.10)^a^ |  |  |  |
| Accommodation | Control (n=30) | 19.20 (17.17 – 21.46) |  |  |  | 0.012 | 0.009 | 0.016 |
|  | Moderate-COVID (n=22) | 18.40 (15.95 – 21.24) | 17.18 (15.13 – 19.51) | 17.90 (15.99– 20.05) | 19.72 (17.48 – 22.25)^b^ |  |  |  |
|  | Severe-COVID (n=18) | 19.83 (16.73 – 23.51) | 24.38 (21.29 – 27.93)^a * #^ | 22.77 (19.76 – 26.24)^a #^ | 24.88 (22.64 – 27.35)^a * #^ |  |  |  |
| Accommodation index | Control (n=30) | 1.97 (1.83 – 2.12) |  |  |  | <0.001 | 0.134 | <0.001 |
|  | Moderate-COVID (n=22) | 2.29 (2.17 – 2.42)^*^ | 2.03 (1.87 – 2.21)^a^ | 1.92 (1.75 – 2.09)^a^ | 1.86 (1.71 – 2.02)^a^ |  |  |  |
|  | Severe-COVID (n=18) | 1.52 (1.31 – 1.75)^* #^ | 1.87 (1.74 – 2.00)^a^ | 1.74 (1.60 – 1.89)^a *^ | 1.81 (1.66 – 1.96) |  |  |  |
| **SET for vastus lateralis** | | | | |  |  |  |  |
| Rheobase | Control (n=30) | 7.87 (7.13 –8.68) |  |  |  | 0.001 | 0.001 | 0.011 |
|  | Moderate-COVID (n=22) | 7.04 (6.12 – 8.08) | 7.74 (6.87 – 8.71) | 8.28 (7.37 – 9.30)^a^ | 8.94 (7.97 – 10.03)^a b^ |  |  |  |
|  | Severe-COVID (n=18) | 8.85 (7.91 – 9.90)^#^ | 9.60 (8.37 – 11.01)^* #^ | 9.58 (8.63 – 10.63)^*^ | 10.11 (9.10 – 11.22)^a *^ |  |  |  |
| Chronaxie (µs) | Control (n=30) | 157.33 (139.92 – 176.91) |  |  |  | <0.001 | 0.006 | 0.002 |
|  | Moderate-COVID (n=22) | 197.72 (171.18 – 228.39)^*^ | 211.36 (186.30 – 239.79)^*^ | 209.09 (182.95 – 238.95)^*^ | 182.72 (153.49 – 217.52) |  |  |  |
|  | Severe-COVID (n=18) | 311.11 (243.34 – 397.75)^* #^ | 244.44 (194.40 – 307.37)^*^ | 212.22 (180.92 – 248.93)^a *^ | 213.88 (159.02 – 287.68)^a^ |  |  |  |
| Accommodation^aa, cc^ | Control (n=30) | 15.43 (13.52 – 17.61) |  |  |  | 0.032 | 0.018 | 0.061 |
|  | Moderate-COVID (n=22) | 15.13 (12.68 – 18.06) | 15.45 (13.68 – 17.45) | 15.27 (13.31 – 17.51) | 16.45 (14.66 – 18.46) |  |  |  |
|  | Severe-COVID (n=18)^**,##^ | 16.50 (14.62 – 18.61) | 18.94 (16.55 – 21.67) | 18.00 (15.77 – 20.53) | 20.44 (17.85 – 23.40) |  |  |  |
| Accommodation index | Control (n=30) | 1.89 (1.78 – 2.01) |  |  |  | 0.780 | 0.214 | 0.007 |
|  | Moderate-COVID (n=22) | 2.10 (1.91 – 2.31) | 2.01 (1.84 – 2.20) | 1.84 (1.67 – 2.01)^a b^ | 1.87 (1.71 – 2.03)^a^ |  |  |  |
|  | Severe-COVID (n=18) | 1.88 (1.71 – 2.05) | 2.01 (1.78 – 2.27) | 1.89 (1.70 – 2.11) | 1.98 (1.84 – 2.13) |  |  |  |
| **SET for vastus medialis** | | | | | |  |  |  |
| Rheobase | Control (n=30) | 6.46 (5.78 – 7.23) |  |  |  | <0.001 | <0.001 | <0.001 |
|  | Moderate-COVID (n=22) | 5.68 (4.76 – 6.77) | 5.77 (5.05 – 6.59) | 6.04 (5.44 – 6.71) | 7.36 (6.63 – 8.17)^a b c^ |  |  |  |
|  | Severe-COVID (n=18) | 8.66 (7.65 – 9.81)^* #^ | 8.77 (7.82 – 9.84)^* #^ | 8.77 (7.73 – 9.95)^* #^ | 10.11 (8.75 – 11.67)^b c * #^ |  |  |  |
| Chronaxie (µs) | Control (n=30) | 148.00 (128.04 – 171.06) |  |  |  | <0.001 | 0.003 | 0.004 |
|  | Moderate-COVID (n=22) | 170.72 (157.13 – 189.86) | 172.72 (157.13 – 189.86) | 188.63 (169.79 – 209.57)^a *^ | 155.45 (139.49 – 173.24)^c^ |  |  |  |
|  | Severe-COVID (n=18) | 647.22 (285.64 – 1466.49) | 208.33 (167.28 – 259.44)^*^ | 241.66 (189.89 – 307.54)^*^ | 202.77 (150.20 – 273.75) |  |  |  |
| Accommodation | Control (n=30) | 12.36 (10.68 – 14.30) |  |  |  | <0.001 | <0.001 | <0.001 |
|  | Moderate-COVID (n=22) | 11.09 (9.11 – 13.28) | 11.31 (9.64 – 13.28) | 11.54 (9.99 – 13.34) | 14.77 (13.03 – 16.74)^a b c^ |  |  |  |
|  | Severe-COVID (n=18) | 18.44 (16.47 – 20.65)^* #^ | 17.66 (15.80 – 19.75)^* #^ | 17.55 (15.78 – 19.52)^* #^ | 19.94 (17.51 – 22.70)^c * #^ |  |  |  |
| Accommodation index | Control (n=30) | 1.87 (1.74 – 2.01) |  |  |  | 0.068 | 0.508 | 0.646 |
|  | Moderate-COVID (n=22) | 1.95 (1.77 – 2.15) | 1.95 (1.79 – 2.13) | 1.87 (1.75 – 2.01) | 1.99 (1.86 – 2.13) |  |  |  |
|  | Severe-COVID (n=18) | 2.18 (1.98 – 2.41) | 2.04 (1.87 – 2.21) | 2.03 (1.90 – 2.16) | 2.03 (1.84 – 2.23) |  |  |  |
| Footnote: CI= confidence interval; n= number of participants; a= different from baseline assessment; b= different from assessment 2; c= different from assessment 3; #= different from moderate-COVID at the same assessment; *= different from the Control; ##= different from moderate-COVID, main effect of group; **= different from control, main effect group; aa=assessment 4 different from baseline assessment, main effect of assessment; cc= assessment 4 different from assessment 3, main effect of assessment; (p<0.05). All outcomes were analyzed were analyzed without a moderator (QIC lowest), except for chronaxie for RF, accommodation index for RF and rheobase for VL that were analyzed considering sex as a moderator (QIC lowest and p < 0.05 for sex). | | | | | | | | |

**Table S3. Total-TTI during fatigue protocol analysis**

|  |  | **Baseline assessment** | **Assessment 2** | **Assessment 3** | **Assessment 4** | **GEE (*p* values)** | | |
| --- | --- | --- | --- | --- | --- | --- | --- | --- |
|  | **Groups** | **Mean (95% CI)** | **Mean (95% CI)** | **Mean (95% CI)** | **Mean (95% CI)** | **Group** | **Assessment** | **Group* Assessment** |
| Total-TTI (Nm.s) | Control (n=30) | 10249.78 (9343.02 – 11244.54) |  |  |  | 0.001 | 0.049 | 0.017 |
|  | Moderate-COVID (n=22) | 9224.15 (8265.60 – 10293.86) | 10440.48 (9420.05 – 11571.45)^a^ | 10397.35 (9522.97 – 11352.02)^a^ | 10478.15 (9506.25 –11549.42)^a^ |  |  |  |
|  | Severe-COVID (n=18) | 6901.27 (5639.32 – 8445.62)^*, #^ | 7861.96 (6679.66 – 9253.52)^*, #^ | 7926.89 (6684.48 – 9400.21)^*, #^ | 7636.97 (6804.35– 8571.47)^*, #^ |  |  |  |
| Footnote: CI= confidence interval; n= number of participants; Total-TTI= total torque-time integral; a= different from baseline assessment intragroup; *= different from the Control assessment; #= different from Moderate-COVID at the same assessment. (p<0.05). Analysis considering sex as a moderator (QIC lowest and p < 0.05 for sex). | | | | | | | | |

**Table S4. Fatigue analysis during knee-extension maximal torque production performed in the fatigue protocol**

|  | **Groups** | **Contractions** | **Baseline assessment** | **Assessment 2** | **Assessment 3** | **Assessment 4** | **GEE *(p* values)** | | | |
| --- | --- | --- | --- | --- | --- | --- | --- | --- | --- | --- |
|  |  |  | **Mean (95% CI)** | **Mean (95% CI)** | **Mean (95% CI)** | **Mean (95% CI)** | **Group * Assessment** | **Group * contraction** | **Assessment * contraction** | **Group * Assessment * contraction** |
| **Fatigue protocol analysis** | | | | | | | | | | |
| TF(%) | Control (n=30) | 1 | 95.36 (92.61 –98.19) |  |  |  | 0.216 | 0.035 | 0.201 | 0.497 |
|  |  | 5^aa^ | 84.14 (80.54 – 87.90) |  |  |  |  |  |  |  |
|  |  | 10^aa,bb^ | 71.98 (67.96 – 76.24) |  |  |  |  |  |  |  |
|  |  | Total | 83.28 (80.36 – 86.30) |  |  |  |  |  |  |  |
|  | Moderate-COVID (n=22) | 1^dd, ee^ | 97.35 (93.64 – 101.21) | 97.62 (95.12 – 100.19) | 97.08 (93.56 – 100.73) | 98.87 (95.72 – 102.11) |  |  |  |  |
|  |  | 5^aa, cc, ee^ | 83.92 (79.40 – 88.71) | 81.58 (77.18 – 86.22) | 82.49 (78.15 – 87.08) | 84.58 (80.34 – 89.06) |  |  |  |  |
|  |  | 10^aa, bb, cc, dd^ | 71.56 (66.80 – 76.66) | 66.81 (62.07 – 71.90) | 70.69 (66.62 – 75.01) | 74.71 (70.33 – 79.37) |  |  |  |  |
|  |  | Total | 83.62 (80.18 – 87.20) | 81.03 (77.70 – 84.50) | 82.72 (79.22 – 86.38) | 85.49 (82.23 – 88.88) |  |  |  |  |
|  | Severe-COVID (n=18) | 1^dd, ee, gg, hh^ | 99.26 (95.68 – 102.97) | 96.66 (93.02 – 100.45) | 97.00 (94.04 – 100.06) | 97.01 (92.45 – 101.80) |  |  |  |  |
|  |  | 5^aa, cc, ee, ff, hh^ | 81.47 (76.29 – 87.00) | 87.01 (82.33 – 91.94) | 78.35 (73.23 – 83.82) | 84.13 (78.60 – 90.05) |  |  |  |  |
|  |  | 10^aa, bb, cc, dd, ff, gg^ | 73.86 (68.65 – 79.46) | 75.87 (71.02 – 81.06) | 72.62 (67.08 – 78.61) | 77.53 (71.47 – 84.11) |  |  |  |  |
|  |  | Total | 84.22 (80.61 – 87.98) | 86.09 (82.44 – 89.91) | 82.03 (78.47 – 85.75) | 85.85 (81.11 – 90.87) |  |  |  |  |
| Absolute torque (Nm) | Control (n=30) | 1 | 158.98 (147.16 – 171.75) |  |  |  | 0.026 | 0.020 | 0.829 | 0.648 |
|  |  | 5^aa^ | 140.17 (128.73 – 152.63) |  |  |  |  |  |  |  |
|  |  | 10^aa, bb^ | 119.15 (109.17 – 130.05) |  |  |  |  |  |  |  |
|  |  | Total | 138.48 (128.04 – 149.76) |  |  |  |  |  |  |  |
|  | Moderate-COVID (n=22) | 1^dd, ee^ | 149.88 (133.63 – 168.10) | 168.50 (152.64 – 185.99) | 163.92 (149.98 – 179.14) | 169.40 (151.64 – 189.24) |  |  |  |  |
|  |  | 5^aa, cc, ee^ | 129.05 (113.91 – 146.20) | 140.44 (126.90 – 155.43) | 138.78 (126.89 – 151.79) | 143.06 (130.56 – 156.75) |  |  |  |  |
|  |  | 10^aa, bb, cc, dd^ | 109.93 (96.71 – 124.94) | 114.95 (103.20 – 128.04) | 118.00 (108.86 – 127.92) | 127.45 (113.95 – 142.54) |  |  |  |  |
|  |  | Total | 128.59 (114.44 – 144.49) | 139.59 (126.78 – 153.71)^c^ | 138.98 (128.11 – 150.78) | 145.63 (131.71 – 161.03)^c^ |  |  |  |  |
|  | Severe-COVID (n=18) | 1^cc, dd, ff^ | 112.41 (95.47 – 132.35) | 119.83 (104.64 – 137.21) | 130.48 (112.26 – 151.64) | 122.55 (109.67 – 136.93) |  |  |  |  |
|  |  | 5^aa, cc, dd, ff, gg^ | 96.52 (82.98 – 112.26) | 106.81 (94.34 – 120.91) | 107.91 (93.45 – 124.59) | 104.81 (92.61 – 118.61) |  |  |  |  |
|  |  | 10^aa, bb, cc, dd, ee, ff, gg, hh^ | 88.13 (78.90 – 98.44) | 96.30 (84.45 – 109.81) | 98.46 (86.45 – 112.14) | 97.21 (84.13 – 112.32) |  |  |  |  |
|  |  | Total | 98.52 (86.70 – 111.94)^**, ##, ⱡ ⱡ ††, ‡‡^ | 107.22 (94.63 – 121.47)^c, **, ##, ⱡ ⱡ, ††, ‡‡^ | 111.50 (97.55 – 127.45)^c, **, ⱡ ⱡ, ††, ‡‡^ | 107.68 (95.46 – 121.46)^**, ##, ⱡ ⱡ, ††, ‡‡^ |  |  |  |  |
| TTI-F (%) | Control (n=30) | 1 | 92.48 (89.67 – 95.38) |  |  |  | 0.755 | 0.011 | 0.513 | 0.236 |
|  |  | 5^aa^ | 74.68 (69.19 – 80.60) |  |  |  |  |  |  |  |
|  |  | 10^aa, bb^ | 63.31 (58.66 – 68.33) |  |  |  |  |  |  |  |
|  |  | Total | 75.90 (72.73 – 79.21) |  |  |  |  |  |  |  |
|  | Moderate-COVID (n=22) | 1^cc, dd, ee^ | 97.21 (93.04 – 101.57) | 96.31 (92.89 – 99.86) | 95.06 (91.16 – 99.14) | 97.47 (94.31 – 100.73) |  |  |  |  |
|  |  | 5^aa, cc, ee^ | 78.13 (73.27 – 83.31) | 77.83 (72.87 – 83.13) | 79.19 (74.11 – 84.63) | 78.75 (73.45 – 84.42) |  |  |  |  |
|  |  | 10^aa, bb, cc, dd^ | 65.11 (60.87 – 69.65) | 59.84 (55.65 – 64.35) | 65.62 (60.15 – 71.59) | 63.25 (58.63 – 68.24) |  |  |  |  |
|  |  | Total | 79.08 (75.95 – 82.34) | 76.55 (73.21 – 80.04) | 79.05 (74.51 – 83.88) | 78.59 (74.80 – 82.59) |  |  |  |  |
|  | Severe-COVID (n=18) | 1^dd,ee, gg, hh^ | 92.52 (83.82 – 102.11) | 92.42 (86.04 – 99.28) | 96.09(92.05 – 100.30) | 94.13 (90.04 – 98.40) |  |  |  |  |
|  |  | 5^aa, cc, ee, ff, hh^ | 73.05 (65.53 – 81.43) | 80.41 (74.77 – 86.47) | 75.56 (71.23 – 80.16) | 76.69 (70.12 – 83.88) |  |  |  |  |
|  |  | 10^aa, bb, cc, ff, gg^ | 69.00 (61.03 – 78.01) | 70.72 (64.63 – 77.37) | 66.58 (60.93 – 72.76) | 69.64 (64.38 – 75.32) |  |  |  |  |
|  |  | Total | 77.55 (72.66 – 82.77) | 80.70 (76.19 – 85.47) | 78.48 (74.40 – 82.79) | 79.51 (74.90 – 84.41) |  |  |  |  |
| Absolute TTI (Nm.s) | Control (n=30) | 1 | 1271.41 (1159.97 – 1393.55) |  |  |  | 0.006 | 0.014 | 0.454 | 0.365 |
|  |  | 5^aa^ | 1027.55 (914.69 – 1154.34) |  |  |  |  |  |  |  |
|  |  | 10^aa, bb^ | 857.50 (771.76 – 952.76) |  |  |  |  |  |  |  |
|  |  | Total | 1038.58 (945.79 – 1140.47) |  |  |  |  |  |  |  |
|  | Moderate-COVID (n=22) | 1^dd, ee^ | 1135.73 (1024.02 – 1259.63) | 1313.95 (1181.01 – 1461.84) | 1246.32 (1134.68 – 1368.94) | 1316.29 (1174.43 – 1475.27) |  |  |  |  |
|  |  | 5^aa, cc, ee^ | 911.73 (807.12 – 1029.90) | 1060.79 (948.86 – 1185.93) | 1025.11 (940.13 – 1117.77) | 1042.11 (948.09 – 1145.45) |  |  |  |  |
|  |  | 10^aa, bb, cc, dd^ | 748.93 (677.64 – 827.72) | 815.57 (728.86 – 912.60) | 846.75 (764.89 – 937.37) | 840.93 (756.97 – 934.21) |  |  |  |  |
|  |  | Total | 918.74 (831.92 – 1014.63) | 1043.66 (942.12 – 1156.13)^c^ | 1026.56 (941.02 – 1119.88)^c^ | 1048.76 (951.64 – 1155.78)^c^ |  |  |  |  |
|  | Severe-COVID (n=18) | 1^cc, ff^ | 884.87 (711.99 – 1099.74) | 925.02 (792.15 – 1080.18) | 1006.91 (866.06 – 1170.66) | 954.19 (838.88 – 1085.35) |  |  |  |  |
|  |  | 5^aa, cc, dd, ff, g^ | 724.04 (601.45 – 871.62) | 789.69 (691.13 – 902.30) | 820.63 (707.17 – 952.29) | 752.03 (655.12 – 863.28) |  |  |  |  |
|  |  | 10^aa, bb, cc, dd, ee, ff,^ ^gg^ | 672.99 (586.66- 772.03) | 718.11 (616.10 – 837.01) | 714.23 (614.51 – 830.13) | 683.26 (599.53 – 778.69) |  |  |  |  |
|  |  | Total | 755.47 (643.05 – 887.55)^**, ##, ⱡ ⱡ, ††, ‡‡^ | 806.49 (702.23 – 926.24)^**, ⱡ ⱡ, ††, ‡‡^ | 838.80 (726.52 – 968.43)^**, ⱡ ⱡ, ††, ‡‡^ | 788.53 (696.93 – 892.18)^**, ⱡ ⱡ, ††, ‡‡^ |  |  |  |  |
| PE | Control (n=30) | Pre fatigue | 9.50 (8.63 – 10.45) |  |  |  | <0.001 | 0.082 | 0.020 | 0.001 |
|  |  | 5 | 15.33 (14.11– 16.66)^a^ |  |  |  |  |  |  |  |
|  |  | 10 | 17.80 (16.70 – 18.96)^a, b^ |  |  |  |  |  |  |  |
|  |  | Total | 13.73 (12.81 – 14.73) |  |  |  |  |  |  |  |
|  | Moderate-COVID (n=22) | Pre fatigue | 10.68 (9.60 – 11.88) | 10.22 (9.02 – 11.59) | 9.77 (8.58 – 11.12) | 8.54 (7.42 – 9.83)^c, d^ |  |  |  |  |
|  |  | 5 | 17.46 (16.48 – 18.48)^a, **^ | 17.13 (15.76 – 18.63)^a^ | 17.27 (16.34 – 18.25)^a, **^ | 16.45 (15.44 – 17.52)^a, c^ |  |  |  |  |
|  |  | 10 | 19.00 (18.38 – 19.64)^a, b^ | 19.18 (17.98 – 20.45)^a, b^ | 19.31 (18.70 – 19.95)^a, b, **^ | 19.22 (18.76 – 19.70)^a, b, **^ |  |  |  |  |
|  |  | Total | 15.24 (14.48 – 16.04) | 14.98 (13.88 – 16.16) | 14.82 (13.93 – 15.77) | 13.93 (13.08 – 14.83) |  |  |  |  |
|  | Severe-COVID (n=18) | Pre fatigue | 12.60 (10.87 – 14.59)^**^ | 9.77 (8.70 – 10.98)^c^ | 10.00 (8.50 – 11.75)^c^ | 10.22 (8.83 – 11.82)^c^ |  |  |  |  |
|  |  | 5 | 17.21 (15.50 – 19.10)^a^ | 15.50 (14.01 – 17.13)^a^ | 15.37 (13.66 – 17.29)^a, c^ | 14.56 (13.05 – 16.23)^a, c, ##^ |  |  |  |  |
|  |  | 10 | 18.69 (17.84 – 19.57)^a, b^ | 17.76 (16.66 – 18.93)^a, b^ | 17.06 (15.40 – 18.89)^a, b, c, ##^ | 17.50 (16.34 – 18.74)^a, b, c, ##^ |  |  |  |  |
|  |  | Total | 15.94 (14.55 – 17.47) | 13.91 (12.96 – 14.93) | 13.79 (12.51 – 15.19) | 13.75 (12.55 – 15.08) |  |  |  |  |
| Relative RMS of knee extensors (%) | Control (n=30) | 1 | 83.96 (79.83 – 88.30) |  |  |  | 0.525 | 0.035 | 0.206 | 0.134 |
|  |  | 5 | 82.65 (76.98 – 88.75) |  |  |  |  |  |  |  |
|  |  | 10^aa, bb^ | 77.13 (70.81 – 84.01) |  |  |  |  |  |  |  |
|  |  | Total | 81.19 (76.49 – 86.18) |  |  |  |  |  |  |  |
|  | Moderate-COVID (n=22) | 1^ee^ | 85.76 (80.10 – 91.82) | 88.90 (83.80 – 94.30) | 82.82 (77.12 – 88.94) | 85.80 (81.20 – 90.66) |  |  |  |  |
|  |  | 5 | 84.72 (75.65 – 94.88) | 84.54 (76.71 – 93.17) | 81.20 (73.98 – 89.13) | 84.61 (79.06 – 90.55) |  |  |  |  |
|  |  | 10^aa, bb, cc, dd^ | 72.89 (64.85 – 81.92) | 72.08 (64.34 – 80.74) | 71.07 (64.11 – 78.79) | 79.38 (73.83 – 85.34) |  |  |  |  |
|  |  | Total | 80.90 (75.06 – 87.20) | 81.52 (75.56 – 87.94) | 78.19 (72.35 – 84.49) | 83.22 (79.37 – 87.25) |  |  |  |  |
|  | Severe-COVID (n=18) | 1^ee, hh^ | 91.86 (84.70 – 99.63) | 86.23 (80.06 – 92.87) | 87.27 (83.22 – 91.51) | 84.73 (78.05 – 91.98) |  |  |  |  |
|  |  | 5^hh^ | 80.20 (71.56 – 89.88) | 89.34 (82.36 – 96.92) | 80.85 (74.46 – 87.79) | 84.27 (75.51 – 94.04) |  |  |  |  |
|  |  | 10 | 80.41 (69.97 – 92.41) | 83.30 (74.26 – 93.44) | 82.64 (71.88 – 95.02) | 83.01 (74.53 – 92.46) |  |  |  |  |
|  |  | Total | 83.92 (77.83 – 90.49) | 86.21 (79.89 – 93.03) | 83.49 (77.23 – 90.26) | 83.95 (77.25 – 91.25) |  |  |  |  |
| mFREQ of knee extensors (Hz) | Control (n=30) | 1 | 82.18 (78.96- 85.53) |  |  |  | 0.002 | 0.363 | 0.951 | 0.879 |
|  |  | 5 | 74.53 (72.06 – 77.08) |  |  |  |  |  |  |  |
|  |  | 10 | 72.32 (69.99 – 74.72) |  |  |  |  |  |  |  |
|  |  | Total | 76.23 (73.88 – 78.65) |  |  |  |  |  |  |  |
|  | Moderate-COVID (n=22) | 1 | 77.74 (73.82 – 81.87) | 80.67 (76.93 – 84.58) | 82.00 (78.71 – 85.42) | 80.84 (78.18 – 83.58) |  |  |  |  |
|  |  | 5 | 70.91 (67.28 – 74.72) | 72.36 (68.83 – 76.07) | 74.16 (70.81 – 77.67) | 73.88 (71.44 – 76.39) |  |  |  |  |
|  |  | 10 | 69.86 (67.13 – 72.70) | 69.78 (66.37 – 73.35) | 71.47 (68.19 – 74.91) | 71.02 (68.81 – 73.30) |  |  |  |  |
|  |  | Total | 72.75 (69.65 – 76.00) | 74.13 (70.77 – 77.64) | 75.75 (72.73 – 78.89)^c^ | 75.13 (73.07 – 77.26)^c^ |  |  |  |  |
|  | Severe-COVID (n=18) | 1 | 74.08 (69.41 – 79.06) | 77.93 (74.50 – 81.51) | 79.17 (74.83 – 83.75) | 78.77 (75.56 – 82.11) |  |  |  |  |
|  |  | 5 | 68.34 (64.52 – 72.39) | 72.41 (69.21 – 75.75) | 73.24 (68.88 – 77.87) | 73.65 (70.36 – 77.10) |  |  |  |  |
|  |  | 10 | 66.56 (64.36 – 68.84) | 70.90 (66.78 – 75.29) | 71.64 (67.48 – 76.06) | 72.74 (69.10 – 76.57) |  |  |  |  |
|  |  | Total | 69.59 (66.40 – 72.93)^**, ††, ‡‡^ | 73.69 (70.32 – 77.21)^c^ | 74.61 (70.54 – 78.92)^c^ | 75.01 (71.89 – 78.26)^c^ |  |  |  |  |
| Footnote: CI= confidence interval; n= number of participants; TF= torque fatigability; RMS= root mean squared; mFREQ= medium frequency; TTI= torque-time integral; TTI-F= Torque-time integral fatigability; PE= Perceived exertion; a= different from contraction 1 intragroup; b= different from contraction 5 intragroup; c= different from baseline assessment intragroup; d=different from assessment 2 intragroup;**= different from the Control; ##= different from baseline assessment of Moderate-COVID; ⱡ ⱡ = different from assessment 2 of Moderate-COVID; ††= different from assessment 3 of Moderate-COVID; ‡‡= different from assessment 4 of Moderate-COVID; aa= different from contraction 1 intragroup, interaction between group and contraction; bb=different from contraction 5 intragroup, interaction between group and contraction; cc=different from contraction 1 of the Control, interaction between group and contraction; dd= different from contraction 5 of the Control, interaction between group and contraction; ee=different from contraction 10 of the Control, interaction between group and contraction; ff= different from contraction 1 of Moderate-COVID, interaction between group and contraction; gg= different from contraction 5 of Moderate-COVID, interaction between group and contraction; hh= different from contraction 10 of Moderate-COVID, interaction between group and contraction. (p<0.05). | | | | | | | | | | |

All outcomes were analyzed were analyzed without a moderator (QIC lowest), except for absolute torque and absolute TTI that were analyzed considering sex as a moderator (QIC lowest and p < 0.05 for sex).
